# Reply to “No pervasive relationship between phyllosphere nitrifier abundance and canopy nitrification in European forests”

**DOI:** 10.1093/nsr/nwag145

**Published:** 2026-03-09

**Authors:** Rossella Guerrieri, Joan Cáliz, Stefania Mattana, Anna Barceló, David Elustondo, Sofie Hellsten, Antti-Jussi Lindroos, Giorgio Matteucci, Päivi Merilä, Manuel Nicolas, Anne Thimonier, Silvia Turroni, Elena Vanguelova, Arne Verstraeten, Peter Waldner, Mirai Watanabe, Emilio O Casamayor, Josep Peñuelas, Maurizio Mencuccini

**Affiliations:** Department of Agricultural and Food Sciences, University of Bologna, Italy; Centre of Advanced Studies of Blanes, CEAB-CSIC, Spanish Council for Scientific Research, Spain; Department of Agri-Food Engineering and Biotechnology, Universitat Politècnica de Catalunya, Spain; Servei de Genòmica i Bioinformàtica, Universitat Autònoma de Barcelona, Spain; Instituto de Biodiversidad y Medioambiente (BIOMA), Universidad de Navarra, Spain; IVL Swedish Environmental Research Institute, Sweden; Natural Resources Institute Finland (Luke), Finland; CNR-IBE, Bologna, Italy; Natural Resources Institute Finland (Luke), Finland; ONF, Office National des Forêts, Departement Recherche et Developpement, France; WSL, Swiss Federal Institute for Forest, Snow and Landscape Research, Switzerland; Department of Pharmacy and Biotechnology, University of Bologna, Italy; Centre for Ecosystem, Society and Biosecurity, Forest Research, UK; Research Institute for Nature and Forest, Belgium; WSL, Swiss Federal Institute for Forest, Snow and Landscape Research, Switzerland; National Institute for Environmental Studies, Japan; Centre of Advanced Studies of Blanes, CEAB-CSIC, Spanish Council for Scientific Research, Spain; CREAF, Spain; CSIC, Global Ecology Unit CREAF-CSIC-UAB, Spain; CREAF, Spain; ICREA, Spain

Forest canopies have long been recognized for their significant role in nitrogen (N) cycling [1–6]. Since the first evidence of nitrifiers colonizing the phyllosphere of Norway spruce back in 2002 [7], nitrification has been discussed as a key biological process in the interaction between atmospheric N and tree canopies [8–12]. However, its relevance at the ecosystem scale has been rarely elucidated. Guerrieri *et al.* [13] (from now on, referred to as G2024) quantified the contribution of biological nitrification to ecosystem N fluxes and demonstrated the presence of nitrifiers in the phyllosphere of *Fagus sylvatica* (*F. sylvatica*) and *Pinus sylvestris* (*P. sylvestris*) forests across a broad environmental gradient in Europe. G2024 found that on average 32% ± 25% of the total NO_3_^−^ in throughfall (TF) derived from canopy nitrification, with a range of the fraction of NO_3_^−^ coming from biological transformation (*f*_Bio_) from 4% to 80%. *f*_Bio_ was higher in *F. sylvatica* than *P. sylvestris*, and it increased with atmospheric N deposition. When accounting for *f*_Bio_ in TF fluxes, G2024 reported that gross canopy nitrification (GCN) added 0.40–4.97 kg N ha^−1^ yr^−1^ (*F. sylvatica*) and 0.21–3.23 kg N ha^−1^ yr^−1^ (*P. sylvestris*) to the NO_3_^−^ from atmospheric deposition.

Recently, Duan *et al.* (this Comment) challenged these findings, based on the re-analyses of data presented in G2024. They found overall a weak relationship between *f*_Bio_ or GCN (estimated from stable oxygen isotopes and N fluxes) and abundance of archaeal and bacterial nitrifiers (from quantitative polymerase chain reaction). The authors identified three limitations in G2024 that could contribute to explaining the lack of the relationship. While the points raised by Duan *et al.* are relevant, we show that they do not provide compelling evidence disproving the occurrence of GCN as quantified in G2024.

An important premise is needed. G2024 quantified the process and its contribution in terms of N flux integrated over the growing season. However, the molecular analyses did not allow ascertainment of when nitrifiers were active in carrying out the process. For instance, nitrifiers may be more active in processing atmospheric N during a dry period, when dry deposition can be retained and it remains on leaf surfaces for longer periods [12]. The mass balance approach based on Δ^17^O in G2024 detects the biologically derived NO_3_^−^ cumulated over days or months, which is washed off the canopies during a precipitation event, thus adding on the atmospheric input of NO_3_^−^ in TF. This can lead to a decoupling between when the process occurs and when it is detected. Hence, it is not surprising that canopy nitrification and nitrifiers abundance show a weak correlation.

## LIMITATION 1: STEMFLOW NEGLECTED IN FOREST ECOSYSTEM FLUXES

Duan *et al.* argued that one of the possible reasons for the weak relationship between GCN and abundance of nitrifiers can be the exclusion of stemflow (SF) flux in G2024. The flux reaching the soil via SF would greatly depend on bark features as well as canopy interception, besides precipitation. For instance, SF in *F. sylvatica* can be higher than in *P. sylvestris*, due to its smoother bark and funnel-shaped crown [14]. We focused on TF as it is generally a higher flux than SF, particularly during summer [3,15] and it is the flux most frequently considered in studies assessing the role of tree canopies in retaining or releasing N [6]. To the best of our knowledge, only one study reported presence of nitrifiers on stem bark [16], but the process itself was not quantified. Even if nitrifiers were present on the bark and nitrification occurred, we would have underestimated GCN, but this does not disprove the occurrence of the process. For example, at sites G2024 observed the highest *f*_Bio_, i.e. *F. sylvatica* in Hoeilaart and *P. sylvestris* in Brasschaat, the long-term (1996–2023) contribution of SF to total precipitation (TF + SF) was 1% and <6.4%, respectively (Verstraeten, personal communication), which is in line with data measured across the ICP Forests Level II sites (17; Waldner *et al.*, personal communication). For *F. sylvatica* in Hoeilaart we could calculate the *f*_Bio_ and GCN for 2016. NO_3_ flux in stemflow was 0.2 kg ha^−1^ yr^−1^ (TF was 7.2 kg ha^−1^ yr^−1^), *f*_Bio_ was 0.17, leading to a GCN of 0.034 kg ha^−1^ yr^−1^. Bacterial nitrifiers in stemflow water (both bacterial *amo*A and bacterial *nxr*B) were present (10^7.3^ and 10^5.1^ copy number per ng of DNA extracted, respectively), while archaeal nitrifiers (*amo*A) were not detected. Again, this may underestimate GCN, but it does not disprove the key message in G2024 that nitrification occurs in the phyllosphere.

## LIMITATION 2: NEGLECTING NITRIFICATION BY FUNGI AND COMAMMOX AND OTHER COMPONENTS IN THE PHYLLOSPHERE

Duan *et al.* suggested that the lack of relationship between GCN and nitrifiers could be due to the exclusion of fungal and comammox nitrifiers as well as of flowers and fruits/cones as phyllosphere components. Assuming fungal and comammox nitrifiers were present and actively involved in nitrification, our study would underestimate the absolute abundance of nitrifiers in the phyllosphere, but again, this argument does not go against the occurrence of the process.

Flowers and fruits/cones are important components of the phyllosphere, but their phenology is shorter compared to the foliage. Flowering and fructification normally occur at the beginning of the growing season [18,19]; particularly in the case of *F. sylvatica*, fructification alternates mast years with low fruit production years [20]. Whether nitrifiers can be present and be active in pine cones and seed capsules remains to be explored. Our experimental design aimed at capturing the biological process at the maximum of canopy cover (in terms of leaf area index) and of host tree physiological activities. Assessing the biological transformation of atmospheric N following the phenology of tree canopies and their components should be the next important step, but it was not the goal of the study in G2024, which focused on spatial rather than seasonal canopy nitrification.

## LIMITATION 3: UNCERTAINTIES RELATED TO THE ISOTOPE APPROACH

The limitation raised regarding the isotope approach neglects key theoretical understanding on isotope fractionation processes involving NO_3_^−^. Even though the 0.52 coefficient may vary in the relationship between δ^17^O and δ^18^O (the range reported in the literature is 0.5–0.53 [21]), it remains that biological transformations are associated with mass-dependent isotope fractionations, which lead to Δ^17^O equal to zero [22]. Whereas atmospheric NO_3_^−^ is mostly related to mass-independent isotope fractionations during photochemical reactions leading to an excess of ^17^O, with Δ^17^O > 0 [22]. In 2015, Guerrieri *et al.* [11] compared the estimate of *f*_Bio_ using both Δ^17^O and δ^18^O. While the magnitudes differed, both methods indicated the occurrence of canopy nitrification. The discussion should rather focus on capturing seasonal changes in atmospheric NO_3_^−^–Δ^17^O and in NO_3_^−^ collected directly on leaf surfaces, which can both strengthen the estimate of *f*_Bio_ and be more directly linked to the abundance of nitrifiers.

We are grateful to Duan *et al.* for the positive comments on G2024 and for highlighting relevant areas of improvements. We welcome additional evidence that supports or challenges the occurrence of canopy nitrification and its relevance in the ecosystem N flux. We hope this discussion will inspire further research into the microbes inhabiting forest canopies and their role in the atmosphere–tree canopy interactions, employing multidisciplinary and multi-scale approaches. There is no doubt that *in situ* (leaf surface) nitrification should be quantified, perhaps under manipulative conditions (e.g. NH_3_ enhancement systems [23] and/or using labeled N and O isotopes) coupled with omics approaches [24,25] based on RNA analyses, to directly link the process to active nitrifiers and their conditions. We conclude that the overall weak relationships found between *f*_Bio_ or GCN and abundance of nitrifiers should not be over-interpreted, since none of three limitations highlighted by Duan *et al.* is likely to challenge our main conclusion.
